# Heteroepitaxial Growth of High Optical Quality, Wafer-Scale van der Waals Heterostrucutres

**DOI:** 10.1021/acsami.1c11867

**Published:** 2021-10-04

**Authors:** Katarzyna Ludwiczak, Aleksandra Krystyna Da̧browska, Johannes Binder, Mateusz Tokarczyk, Jakub Iwański, Bogusława Kurowska, Jakub Turczyński, Grzegorz Kowalski, Rafał Bożek, Roman Stȩpniewski, Wojciech Pacuski, Andrzej Wysmołek

**Affiliations:** † Faculty of Physics, University of Warsaw, ul. Pasteura 5, 02-093 Warsaw, Poland; ‡ Institute of Physics Polish Academy of Sciences, Al. Lotników 32/46, 02-668 Warsaw, Poland

**Keywords:** layered materials, transition metal dichalcogenides, epitaxy, metalorganic vapor phase epitaxy, molecular beam epitaxy, Raman spectroscopy

## Abstract

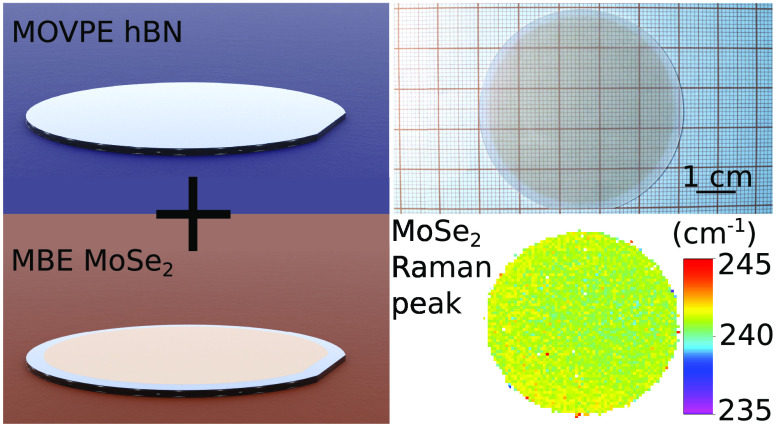

Transition metal dichalcogenides (TMDs) are materials that can exhibit intriguing optical properties like a change of the bandgap from indirect to direct when being thinned down to a monolayer. Well-resolved narrow excitonic resonances can be observed for such monolayers although only for materials of sufficient crystalline quality and so far mostly available in the form of micrometer-sized flakes. A further significant improvement of optical and electrical properties can be achieved by transferring the TMD on hexagonal boron nitride (hBN). To exploit the full potential of TMDs in future applications, epitaxial techniques have to be developed that not only allow the growth of large-scale, high-quality TMD monolayers but also allow the growth to be performed directly on large-scale epitaxial hBN. In this work, we address this problem and demonstrate that MoSe_2_ of high optical quality can be directly grown on epitaxial hBN on an entire 2 in. wafer. We developed a combined growth theme for which hBN is first synthesized at high temperature by metal organic vapor phase epitaxy (MOVPE) and as a second step MoSe_2_ is deposited on top by molecular beam epitaxy (MBE) at much lower temperatures. We show that this structure exhibits excellent optical properties, manifested by narrow excitonic lines in the photoluminescence spectra. Moreover, the material is homogeneous on the area of the whole 2 in. wafer with only ±0.14 meV deviation of excitonic energy. Our mixed growth technique may guide the way for future large-scale production of high quality TMD/hBN heterostructures.

## 1 Introduction

Transition metal dichalcogenides (TMDs), representatives of 2D layered materials, are intensively studied as promising candidates for future realizations of optoelectronic devices,^1,2^ photodetectors,^3−5^ sensors,^6−8^ energy and memory storages,^9−11^ or transistors.^12−14^ Such realizations are commonly demonstrated on micrometer-sized flakes obtained by mechanical exfoliation from bulk crystals and consecutively stacked to a heterostructure by time-consuming deterministic transfer processes.^1,15−18^ This approach allows the production of high quality samples in terms of electronic properties such as carrier mobility or conductivity.^19^

Two-dimensional materials are, however, extremely sensitive to interlayer interactions and prone to environmental factors. Thus, it is important to deposit these materials on flat substrates with homogeneous dielectric properties and without dangling bonds. Hexagonal boron nitride (hBN) appears to be the perfect candidate for this purpose. The encapsulation in thin layers of hBN can significantly improve optical properties of the flakes and prolong their lifetime.^20−24^

The next necessary step toward the practical utilization of the properties of TMDs requires, however, large-area sample synthesis. Significant efforts have been made to demonstrate wafer-scale growth.^25,26^ Approaches based on CVD (chemical vapor deposition) and PVD (physical vapor deposition) techniques result in promising large area monolayers^27−32^ but excellent optical quality could be only achieved after additional postgrowth processing by a sequence of transfer and encapsulation.^33,34^

Epitaxial method like metalorganic vapor phase epitaxy (MOVPE) was also used to address the TMD scalability problem^35^ and it has been shown that it is possible to produce wafer-scale materials although so far not with the same optical properties. Molecular beam epitaxy (MBE) appears to be a promising candidate for the development of large-area samples, as it allows the production of high purity materials with atomic precision.^36−42^ However, the optical quality of the samples is still far behind the best results obtained for mechanically exfoliated flakes. A way to overcome this obstacle is to combine TMD layers with hBN. Successful realizations of this approach such as MoSe_2_ growth by MBE or CVD on initially exfoliated hBN flakes^43,44^ or an entirely grown hBN/MoS_2_ heterostructure by CVD^45^ have been already presented.

**Figure 1:**
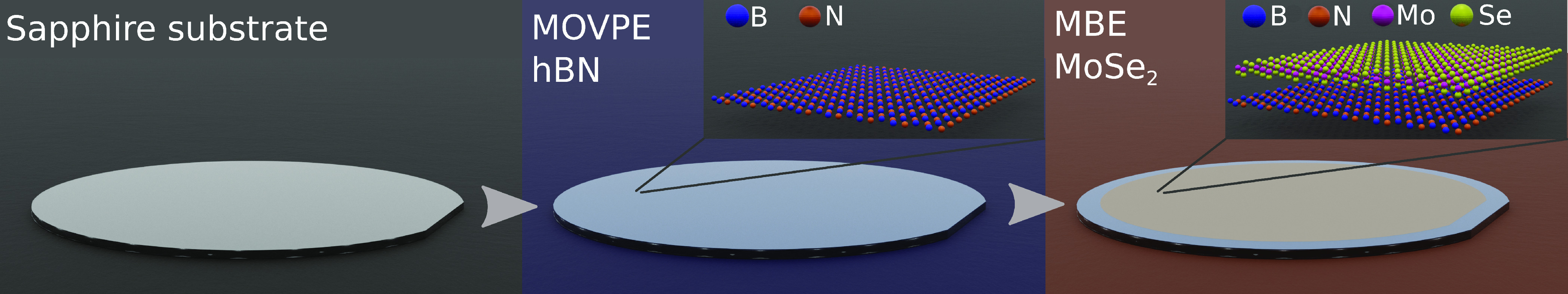
Schematic illustration of the combined growth approach. A 2 in. sapphire wafer is used as a substrate for MOVPE hBN and MBE MoSe_2_ growth.

Here, we present a method that combines two epitaxial techniques: MBE and MOVPE to provide large-scale crystals of high optical quality. We first use MOVPE to grow hBN layers of a few nanometers thickness on a 2 in. sapphire wafer.^46^ Subsequently, we use this sapphire/hBN wafer as a substrate to grow a monolayer of MoSe_2_ by MBE. A schematic illustration of the growth sequence is presented in Figure 1.

The quality of the TMDs is revealed by performing optical measurements such as photoluminescence and Raman spectroscopy. In particular, photoluminescence is a very sensitive probe of layer thickness, because materials like MoS_2_, MoSe_2_, WS_2_, WSe_2_, and MoTe_2_ exhibit an indirect–direct band gap transition, which results in a bright and intense photoluminescence from monolayers in contrast to photoluminescence from thicker samples.^47−50^

Low-temperature photoluminescence spectra are a suitable indicator of the optical quality of MoSe_2_, as excitonic lines can be resolved into two components, corresponding to the neutral A exciton and trion, only for high-quality samples.^51^ In our work, we show that the grown material is of excellent, homogeneous optical quality, confirmed by Raman spectroscopy and photoluminescence mapping of the 2 in. wafer. We demonstrate the unique result of well-resolved excitonic lines at low temperatures on the whole wafer. The proposed approach provides a reliable template for the growth of high optical performance TMD layers by utilizing large area epitaxial hBN as a substrate. Our method of combined MBE–MOVPE growth may pave the way for future applications of van der Waals heterostructures on the wafer scale.

## 2 Results and Discussion

### 2.1 Growth and Characterization of hBN/MoSe_2_ Heterostructures

Hexagonal boron nitride (hBN) layers were grown on 2-in. sapphire wafers by MOVPE. The properties of the obtained hBN layers depend on a set of growth parameters including growth time, temperature, III–V ratio, or the employed growth mode.^52^ One of the key parameters in designing van der Waals heterostructures is the thickness of the hBN spacers. In this work, we study the impact of hBN layer thickness on the properties of the subsequently grown MoSe_2_ monolayer. To be able to obtain hBN layers of different thicknesses, we employed two different growth modes: continuous flow growth (CFG) and a two stage epitaxy to obtain three different hBN samples. CFG growth mode yields ultrathin high-quality hBN layers but shows a self-limiting behavior.^53^ Therefore, this mode cannot be used to obtain samples thicker than a few nanometers. The grown material shows good optical and structural properties with thicknesses ranging from about 1 to 6 nm.

To overcome the obstacle of limited thicknesses, we employ the recently introduced two stage epitaxial growth,^46^ which is a two-step mode for which first a high-quality CFG layer is grown followed by a pulsed growth step that requires an alternate switching of ammonia and TEB flows. With this mode, it becomes possible to grow thicker hBN layers of exceptionally good structural quality (samples hBN1, hBN2).^46^ Samples grown using two stage epitaxy can reach thicknesses from several to tens of nanometers with a lattice constant close to the theoretical value of 3.33 Å.^46^ The growth parameters of the studied samples are summarized in Table 1.

**Table 1:** MOVPE and MBE Growth Parametres of the Samples Presented in This Study^a^

MOVPE		
sample	thickness	growth mode
hBN1	13.6 nm	two stage epitaxy
hBN2	3.5 nm	two stage epitaxy
hBN3	1.5 nm	CFG

MBE	
sample	time of growth
MoSe_2_A	15 h
MoSe_2_B	5 h
MoSe_2_C	5 × 1 h

a In each MBE process, about 1 ML MoSe_2_ is depostied, but the growth rate is 3 times slower in case of the first process (A).

**Figure 2:**
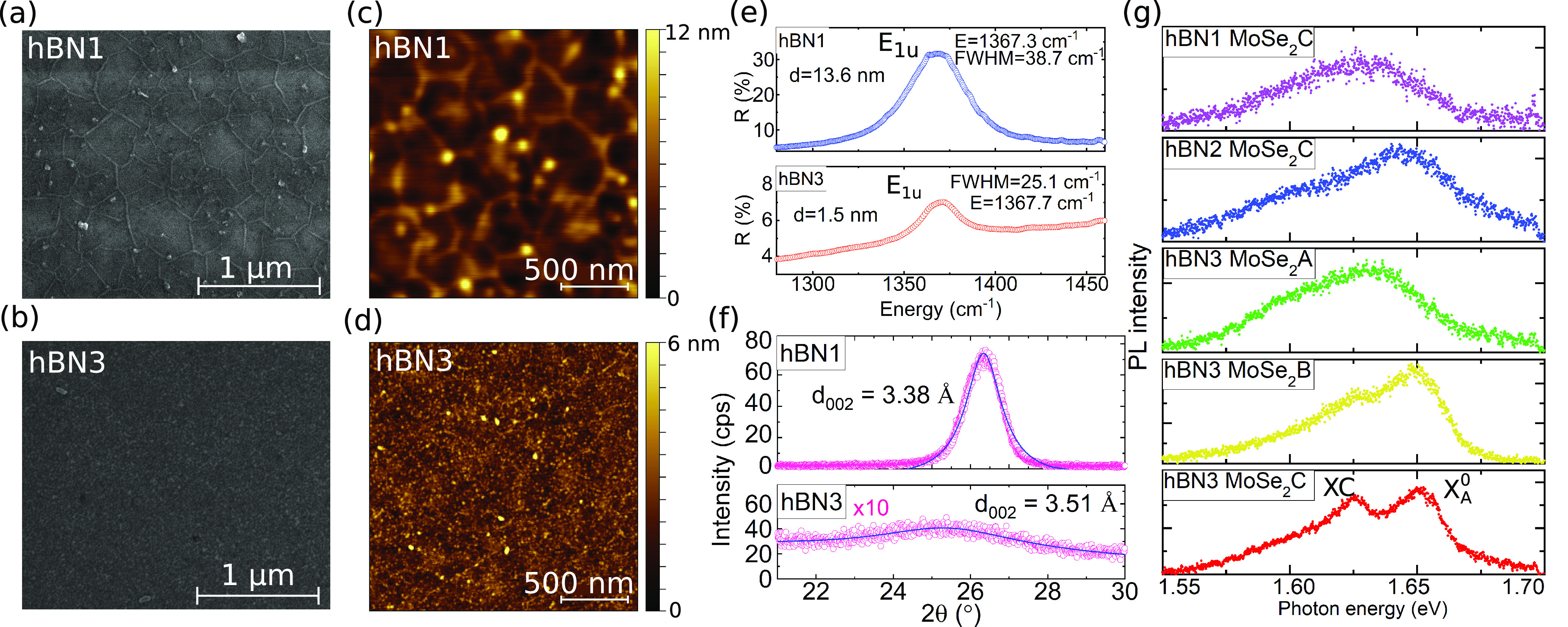
Characterization of hBN substrates obtained in different growth modes. SEM images of (a) hBN1, (b) hBN3; AFM images of (c) hBN1, (d) hBN3 show the topography of the samples: the thinner the hBN layer is, the smaller the precipitations and wrinkles are. (e) FTIR spectra for hBN samples: E_1u_ mode with corresponding energy and thickness. (f) Results of a 2θ/ω scan of the samples with extracted interplanar spacing. (g) Low-temperature photoluminescence spectra obtained for various hBN substrates and MBE growth processes.

Figure 2 presents SEM and AFM images showing the morphology of the thickest (hBN1) and thinnest (hBN3) samples. The MOVPE growth of hBN at high temperature is unavoidably connected to the appearance of characteristic wrinkles on the material. Their presence can be attributed to the postgrowth cooling process and different thermal expansion coefficients of sapphire and hBN. This wrinkle pattern shows a larger mesh size in the case of thicker samples. Other typical objects on the surface of the material are three-dimensional precipitates. Their appearance is most probably the result of the formation of out-of-plane nucleation centers. There is a strong dependence of the precipitates size. The thinner the sample, the smaller the three-dimensional precipitates.

To measure the layer thickness and further characterize the hBN, we performed infrared reflectance spectra measurements using a FTIR microscope and TEM imaging (see Supporting Information Figure S1). By analyzing the spectra within the Dynamic Dielectric Function approximation, we were able to determine the thicknesses of the hBN layers (Figure 2e).^46^ The characteristic peak in the FTIR spectra corresponds to the E_1u_ mode of hBN around 1367 cm^–1^ confirming the formation of a sp^2^-BN layer.^54^

X-ray diffraction (XRD) was used to investigate the structural quality of the samples. The XRD results are presented in Figure 2f. The peak position of around 26.3° for sample hBN1 corresponds to hBN with some turbostratic addition. The peak observed for the thinner sample is less intense and broader, and its angular position of 24.7° suggest mostly turbostratic stacking.^55^

The hBN samples with various thickness were used as substrates for the consecutive growth of MoSe_2_ by MBE. Various growth processes were performed to optimize monolayer formation. We studied the effect of different growth times (growth rates) as well as using one or several annealing steps.

Figure 2g depicts low-temperature photoluminescence spectra for MoSe_2_ layers grown with various MBE processes (MoSe_2_A, MoSe_2_B, MoSe_2_C) and on various epitaxial hBN substrates (hBN1, hBN2, hBN3). At low temperatures, the photoluminescence spectrum of MoSe_2_ unveils additional information about the optical quality of the material. For high-quality samples, the excitonic line separates into two well-resolved peaks corresponding to the A exciton and charged exciton (trion).

Such spectra were mostly observed for mechanically exfoliated material onto selected substrates,^20,56−58^ but recently high optical quality samples were also obtained for MBE grown MoSe_2_ on exfoliated hBN.^44^

The analysis of low-temperature PL spectra of different samples (Figure 2g) allows us to optimize the process of heteroepitaxial growth. The growth parameters for the MoSe_2_A sample were adopted from previous experiments on exfoliated hBN.^44^ This starting point of parameters resulted in a broad spectrum, which can indicate the formation of more than a single atomic layer. The reduction of the growth time for sample MoSe_2_B resulted in a notable improvement of the optical quality of the material and a visible separation of excitonic lines into two peaks (A exciton and trion). A further introduction of several annealing steps into the process resulted in an even clearer separation of the exciton and trion peaks for sample MoSe_2_C. This growth process was performed on three different hBN substrates (hBN1, hBN2, hBN3) during the same growth run. It can be seen that the thinner the hBN layer is, the higher the optical quality of the material is.

**Figure 3:**
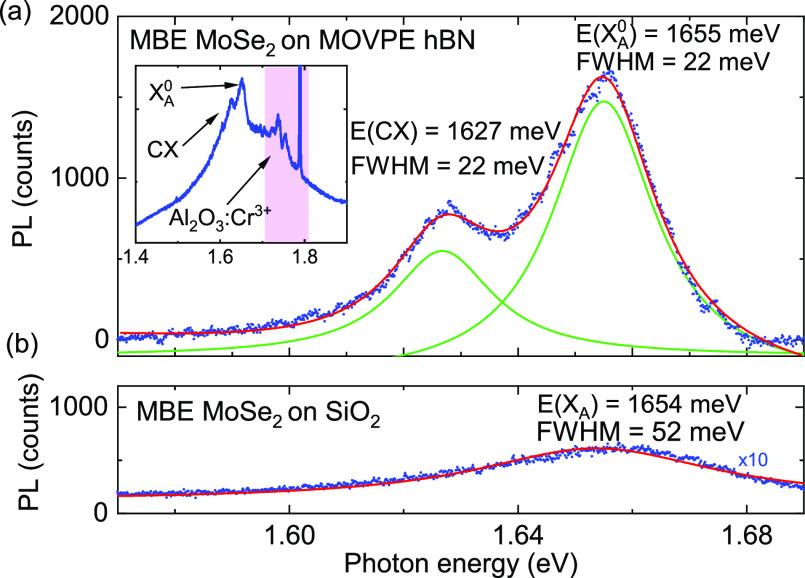
Low-temperature (5 K) photoluminescence spectra of MoSe_2_ grown by MBE on a MOVPE grown hBN compared to material grown by the same MBE process directly on a silicone dioxide substrate. (a) Photoluminescence spectrum of MoSe_2_ grown using optimized MBE and MOVPE processes. The lines of the neutral A exciton (X_A_^0^) and charged exciton (XC) can be resolved. Blue dots show the data, while red and green curves show results of Lorentzian fits. (Inset) photoluminescence spectrum measured in a larger spectral range showing an additional background which emerges from the defects in hBN. (b) Photoluminescence spectrum obtained for MBE MoSe_2_ grown on a SiO_2_ substrate. Excitonic lines are not resolved and the line width of the neutral exciton around 1654 meV is significantly larger (fwhm 52 meV) as compared to the material grown on MOVPE hBN.

The inset in Figure 3a shows a typical spectrum obtained for MoSe_2_C processes grown on hBN3 MOVPE samples in a larger spectral range. The additional background stems from the photoluminescence of defects in the hBN layer. The peaks around 1.7–1.8 eV correspond to the presence of chromium in the sapphire substrates.^59^Figure 3a depicts two distinct peaks typical for MoSe_2_ corresponding to the neutral A exciton and trion with the subtracted background. Peaks are visible at energies of 1655 and 1627 meV, respectively. These results correspond well with the excitonic complexes reported in previous works.^20,56,60,61^ The peak width for the A exciton and trion is 22 meV, which is significantly smaller than the peak width measured for the MoSe_2_ grown on a SiO_2_ substrate Figure 3b and only a factor of 2 larger than for MoSe_2_ grown on flakes exfoliated from high-quality bulk hBN.^44^

**Figure 4:**
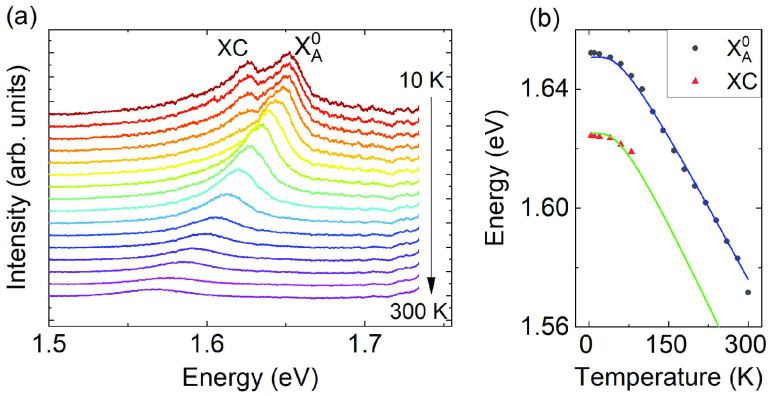
(a) Temperature dependence of the photoluminescence of a MoSe_2_/hBN sample. The spectra were shifted vertically for clarity. At *T* = 10 K, the spectra consist of both the charged and neutral excitonic lines. The charged exciton contribution disappears at around 100 K. (b) Temperature dependence of the energy of the charged and neutral exciton. The blue and green curves present a typical temperature dependence of the excitonic lines of MoSe_2_. The parameters of the curves were taken from ref (56).

The temperature dependence of the excitonic resonances observed in our samples is typical for monolayer MoSe_2_. Figure 4a depicts the evolution of the photoluminescence of the neutral exciton A and trion as a function of temperature (laser excitation wavelength λ = 532). At low temperatures, we observe a binding energy of about 30 meV. The trion signal abruptly decays at around 100 K. Both peaks shift toward lower energies as the temperature rises, due to the reduction of the band gap, shown in Figure 4b.^56,62^ The blue and green lines are not fits to our data but show the typical band gap energy dependence with parameters taken from ref (56) indicating that our material indeed behaves like typical MoSe_2_ without significant influence of stress due to the interaction with hBN layer.

### 2.2 Wafer-Scale Growth of a hBN/MoSe_2_ Heterostrucutre

**Figure 5:**
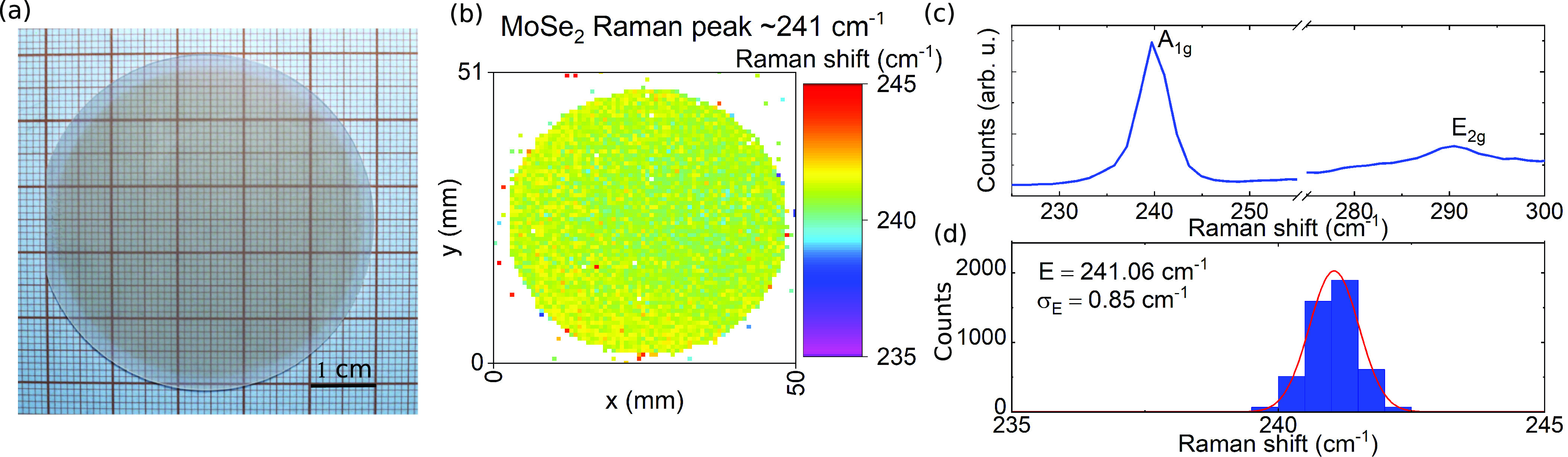
(a) Picture of an epitaxially grown 2 in. hBN/MoSe_2_ van der Waals heterostructure. The visibly darker reddish color indicates MoSe_2_ coverage. (b) Peak energy of the A_1g_ mode obrained by Raman spectroscopy mapping at room temperature. (c) Typical Raman spectrum of the two characteristic MoSe_2_ peaks. (d) Distribution of the peak energy with a mean value of *E*_A_1*g*__ = 241.06 cm^–1^ and standard deviation σ_E_ = 0.85 cm^–1^. The results from the Raman map analysis show that the whole area of the sample is uniformly covered by a single layer of the MoSe_2_.

To further prove that our method can be scaled up, we chose the best MOVPE and MBE processes and grew the layers on an entire 2 in. sapphire substrate. Figure 5a presents a picture of the 2 in. wafer after hBN and MoSe_2_ growth. The material can be observed with the naked eye. The MoSe_2_ layer covers the sample in a circular, visibly darker, brownish area. The transparent, uncovered outer ring is the result of applying a frame that holds the substrate in the MBE chamber. It also allows one to examine the properties of hBN after the MBE growth.

The sample was further studied using Raman spectroscopy. Figure 5b presents the outcome of room-temperature Raman spectroscopy mapping of the whole wafer. The out-of-plane A_1g_ mode of MoSe_2_ at 241 cm^–1^ was chosen for the map analysis, as it is more intense than the in-plane E_2g_ peak (Figure 5c). The presented map confirms that the material indeed uniformly covers the whole area of the sample. Raman spectroscopy is also an excellent tool to assess the number of MoSe_2_ layers.^49,63^ As compared to the bulk material the out-of-plane mode softens (redshifts) while the in-plane mode stiffens (blueshifts) with decreasing thickness. A typical Raman spectrum for this sample is presented in Figure 5c. The highlighted Raman peak positions indicate the presence of a single layer of MoSe_2_. While the peak energy can also vary due to different substrates and exfoliation/growth techniques,^64,65^ the mean value of the A_1g_ peak energy of *E*_A_1*g*__ = 241.06 cm^–1^ for the whole area of the sample with a standard deviation as small as σ_E_ = 0.85 cm^–1^ indeed indicates the formation of a monolayer material. The distribution of the peak energy quantitatively depicted in Figure 5d additionally shows the homogeneity of the material.

**Figure 6:**
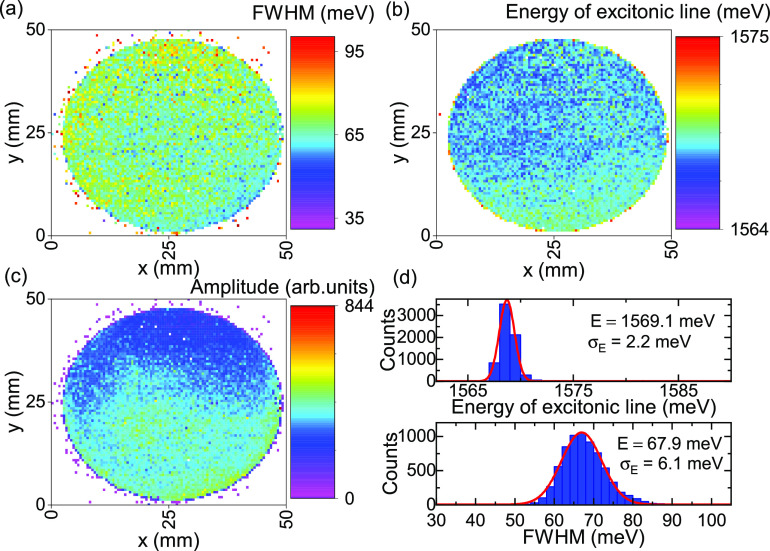
Photoluminescence mapping at room temperature (500 μm step, 633 nm laser). Panels a–c show results of Lorentzian fits to the obtained spectra. A peak width of 67.9 ± 6.1 meV and an energy of 1569.1 ± 2.1 meV for the excitonic line were obtained. The low standard deviation indicates sample homogeneity across the whole 2 in. wafer. (d) Histograms of the excitonic line energy (top) and width (bottom).

Further evidence for the presence of monolayer MoSe_2_ can be provided by photoluminescence mapping. The bandgap becomes direct in the monolayer limit as there are no more vdW interlayer interactions^66^ rendering photoluminescence a very sensitive tool with regard to thickness fluctuations. At room temperature, such a spectrum consists of one distinct peak corresponding to the neutral A exciton with an energy of 1.57 eV for monolayer and 1.54 eV for bilayer MoSe_2_.^49^Figure 6a–d depicts the results of PL mapping of the whole wafer at room temperature. The extracted peak width (Figure 6a) is uniform across the sample with a mean value of 68 ± 6 meV, which is very low for spectra obtained at room temperature.^56^ The energy of the excitonic line does not vary much between different positions on the sample. Its mean value of 1569.1 ± 2.2 meV corresponds perfectly to previously reported results for monolayer MoSe_2_.^49^ Another important factor in determining the thickness of the material is the photoluminescence intensity. It decreases usually by 1 order of magnitude with every additional layer. In our case, the intensity is homogeneous over the whole area of the sample (Figure 6 c). Transmission electron microscope (TEM) characterization of the final sample is in agreement with the optical studies indicating the formation of a MoSe_2_ monolayer (see Supporting Information Figure S1). Further characterization of the final sample morphology is provided by AFM (see Supporting Information Figure S2).

**Figure 7:**
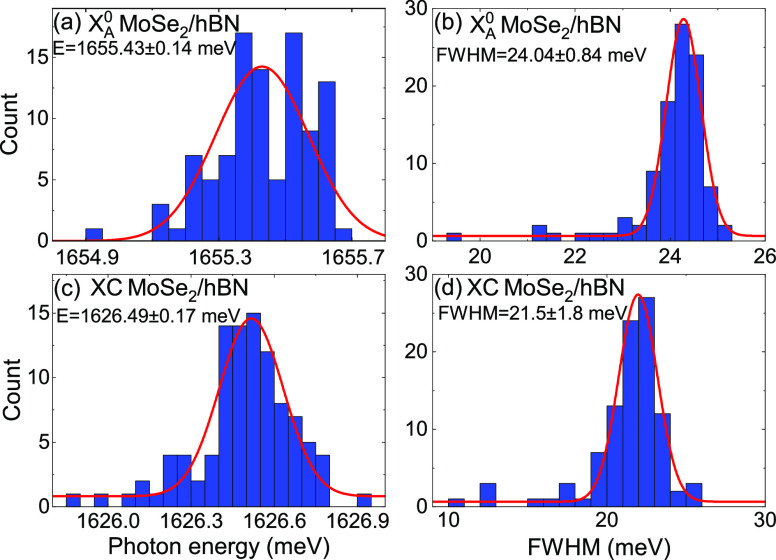
Photoluminescence peaks statistics at *T* = 5 K. The spectra were measured along a line of 1 cm. The histograms show the result of Lorentzian fits to the neutral (X_A_^0^) and charged exciton (XC). (a) distribution of the energy of X_A_^0^. (b) Distribution of peak width of X_A_^0^. (c) Distribution of the energy of XC. (d) Distribution of peak width of XC.

Figure 7a–d depicts the distributions of the peak energy of the A exciton and trion as well as their widths acquired from mapping the sample at liquid helium temperature. Because of the limited size of the cryostat window, we measured 100 spectra uniformly along a straight line (1 cm). A mean energy of 1655.43 ± 0.14 and 1626.49 ± 0.17 meV was extracted for the peaks corresponding to the A exciton and trion, which confirms the large homogeneity of the sample. This aspect is especially important for any future industrial application of TMDs requiring reproducible results, such as a well-defined band gap energy.

## 3 Conclusions

We present a method to produce wafer-scale monolayer TMD samples of high optical quality. Our method combines two epitaxial growth methods: MOVPE, which enables us to grow hBN on a sapphire substrate and MBE which was used to cover the so-grown hBN layer with a monolayer of MoSe_2_. A thorough analysis of the optical properties examined by photoluminescence and Raman spectroscpy mapping reveals a large homogeneity of the epitaxially grown van der Waals heterostructure on the whole 2 in. wafer. We also demonstrate that well-resolved and narrow excitonic peaks at low temperature can be achieved even for such large heteroepitaxial samples. The uniform energy distributions with small standard deviations indicate an excellent homogeneity and repeatability, which is very difficult to achieve with other techniques like mechanical exfoliation. Our results constitute a large step toward a possible industrial implementation of van der Waals heterostructures by enabling wafer-scale production with precisely defined properties.

## 4 Experimental Section

### 4.1 hBN Growth by MOVPE

An Aixtron close coupled showerhead 3 × 2″ system with ARGUS thermal mapping device was used to grow hBN samples. Sapphire wafers (2 in.) purchased from ProCrystal were used as a substrate. Triethylboron (TEB) and ammonia (NH_3_) were used as boron and nitrogen precursors with H_2_ as the carrier gas. Two growth modes were applied to produce the samples discussed in this work:

- Continuous flow growth (sample hBN3): Precursors are simultaneously injected to the reactor at the same time at high temperatures around 1300 °C with supersaturation of ammonia.^46,52^ Such a mode exhibits a self-limiting behavior. The obtained samples show excellent optical quality but cannot be grown thicker than ∼6 nm.
- Two step epitaxy (samples hBN1, hBN2):^46^ The growth of a CFG buffer is followed by a pulsed growth step requiring the switching of ammonia and TEB flows. The obtained samples exhibit excellent structural quality; this way, thicker layers than ∼6 nm can be grown.

### 4.2 MoSe_2_ Growth by MBE

A MBE machine fabricated by SVT Associates was used to grow MoSe_2_ layers. To optimize the process, three hBN samples (hBN1, hBN2, hBN3) were used as substrates for subsequent MBE growth.

- Sample MoSe_2_A was grown in three growth steps, each of which lasted for 5 h and was followed by 20 min of heating up to 700 °C and 10 min annealing in Se flux.
- Sample MoSe_2_B was grown just for 5 h (with 3 times higher growth rate) with only one annealing step at the very end of the process. This time the temperature of the sample was slowly increased up to 700 °C and the annealing in the Se flux lasted for 2 h.
- Sample MoSe_2_C was grown in five consecutive cycles. Each step contained 1 h of growth at 300 °C, a slow heating up to 760 °C and 10 min annealing in Se flux. The last annealing was however significantly longer and lasted for 2 h.

The selection of growth parameters resulted from previous experiences with MoSe_2_ growth on exfoliated hBN as well as optimization to achieve the best optical quality of the samples.

### 4.3 Characterization of the Samples

For low-temperature (5 K) measurements, samples were placed in liquid helium continuous flow cryostat (MicrostatHires Oxford Instruments). Luminescence spectra at ∼5 K were measured with a HORIBA T64000 spectrometer with Nd:YAG 532 nm continuous wave laser.

Raman and photoluminescence mapping of the sample at room temperature was performed with Renishaw inVia spectrometers. The material was excited by a λ = 532 nm laser with a power of 320 μW and a 20× objective (NA 0.4) for Raman mapping and by λ = 633 nm laser, 550 μW laser power, and a 20× objective (NA 0.4) for photoluminescence mapping.

XRD measurements were performed using a Panalytical X’pert diffractometer equipped with Cu X-ray tube.

FTIR reflectance spectra were obtained by Thermo Fischer Scientific iS50, Nicolet Continuum and analyzed within the Dynamic Dielectric Function approximation.

SEM images were acquired using FEI Helios NanoLab 600 and AFM images were acquired by Digital Instruments MMAFM-2 atomic force microscopy.

The morphology and structure of MoSe_2_/BN on sapphire were investigated using a FEI Titan 80-300 transmission electron microscope operating at 300 kV, equipped with an image corrector. The electron transparent cross sections of the heterostructure were cut using a focused gallium ion beam with a Helios Nanolab 600 focused ion beam (FIB) system. Prior to the ion cutting, a gold cap was deposited by magnetron sputtering, which was necessary to eliminate sample charging during TEM lamella preparation.

## Supporting Information Available

TEM image of the final hBN/MoSe_2_ sample; AFM characterization of the final hBN/MoSe_2_ sample morphology (PDF)
