# Supplementary material for: Heteroepitaxial Growth of High Optical Quality, Wafer-Scale van der Waals Heterostrucutres
Source: ACS Appl Mater Interfaces. 2021 Oct 4;13(40):47904–11. doi: 10.1021/acsami.1c11867 (PMC8517960; doi:10.1021/acsami.1c11867)
Supplement: Supplementary file 1 — am1c11867_si_001.pdf [file am1c11867_si_001.pdf]

Supporting Information
Heteroepitaxial growth of high optical quality, wafer-scale van der Waals heterostrucutres

Katarzyna Ludwiczak,[*,†] Aleksandra Krystyna Dąbrowska,[†] Johannes Binder,[†] Mateusz Tokarczyk,[†] Jakub Iwański,[†] Bogusława Kurowska,[‡] Jakub Turczyński,[‡] Grzegorz Kowalski,[†] Rafał Bożek,[†] Roman Stępniewski,[†] Wojciech Pacuski,[†] and Andrzej Wysmołek[†]

†*Faculty of Physics, University of Warsaw, ul. Pasteura 5, 02-093 Warsaw*

‡*Institute of Physics Polish Academy of Sciences, Al. Lotników 32/46, 02-668 Warsaw*

E-mail: kw.ludwiczak@uw.edu.pl

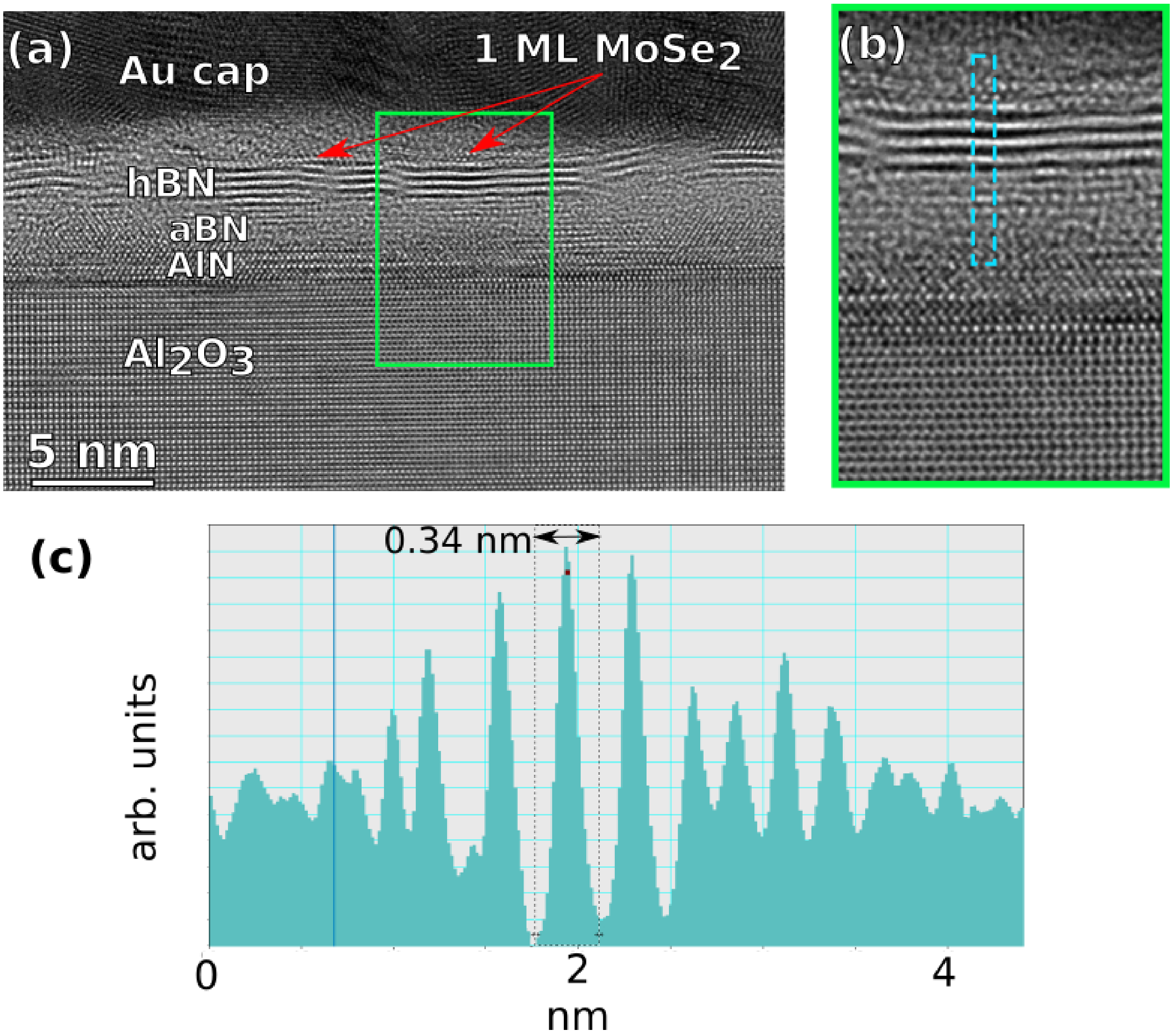

Figure S1: (a) Transmission Electron Microscope image of a hBN/$MoSe_2$ heterostructure (growth processes hBN3, $MoSe_2$C). The hBN layer has a thickness of about 1 nm, which is in good agreement with the FTIR measurements for sample hBN3. At the interface close to sapphire, layers of AlN followed by amorphous BN (aBN) are visible which is typical for MOVPE growth.[1] (b) shows a zoom into the area marked by a green rectangle. The area indicated by a blue rectangle in (b) was used to extract the intensity graph shown in (c). The interlayer spacing extracted from the figure shown in (c) is slightly larger than the literature value of 0.33 nm for hBN. This observation is in agreement with the XRD results presented in the main text that suggest a mostly turbostratic layer stacking in the case of very thin BN layers. The curvature of the atomic planes of BN results from the reproduction of the roughness of the nitrided sapphire surface. The $MoSe_2$ monolayer also reproduces the shape of BN layers and shows 3D bending. For this reason, it is difficult to obtain an image of a long segment of monolayer $MoSe_2$. In addition, the deposition of an Au cap, applied to eliminate charging effects, can partially destroy this layer. The high contrast related to the high atomic number and crystalline structure of gold make a visualization of the $MoSe_2$ monolayer difficult.

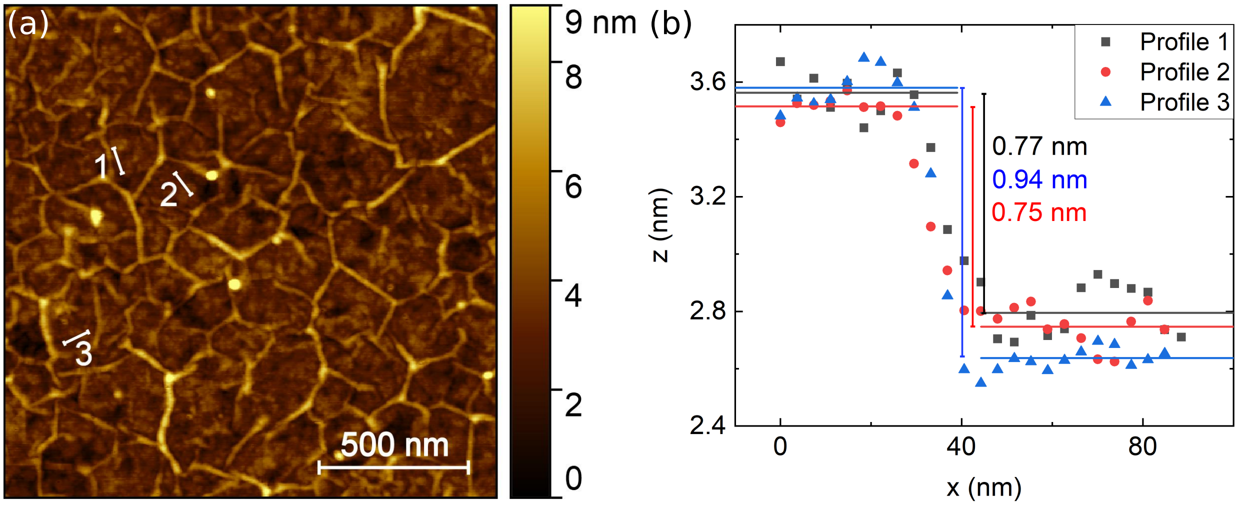

Figure S2: (a) Atomic Force Microscope image of the final hBN/$MoSe_2$ sample. Due to the difference of thermal expansion coefficients between the sapphire substrate and hBN, a characteristic wrinkles pattern appears. The morphology prevents to reliably determine the formation and number of $MoSe_2$ layers. (b) Thickness profiles corresponding to the lines shown in (a). It is possible to find areas on the sample's surface for which the extracted profiles correspond to steps of monolayer $MoSe_2$.

References

(1) Dąbrowska, A. K.; Tokarczyk, M.; Kowalski, G.; Binder, J.; Bozek, R.; Borysiuk, J.; Stępniewski, R.; Wysmołek, A. Two stage epitaxial growth of wafer-size multilayer h-BN by metal-organic vapor phase epitaxy - A homoepitaxial approach. 2D Materials **2020**, 8, 15017.
